# *Notes from the Field*: Outbreak of Cryptosporidiosis Among Collegiate Swimmers and Evidence of Secondary Transmission — Massachusetts and Rhode Island, 2023

**DOI:** 10.15585/mmwr.mm7226a7

**Published:** 2023-06-30

**Authors:** Geena Chiumento, Anthony Osinski, Kelsey DeVoe, Amelia Houghton, Akita Joshi, Caryn Ivanof, Emma Creegan, Michael Gosciminski, Alexandra P. Newman, Susan Madison-Antenucci, Michele C. Hlavsa, Erin Imada, Colleen Lysen, Shanna Miko, Jordan Schultz, Emily Harvey, Johanna Vostok, Catherine M. Brown

**Affiliations:** ^1^Massachusetts Department of Public Health; ^2^College of the Holy Cross, Worcester, Massachusetts; ^3^Worcester Division of Public Health, Central Massachusetts Regional Public Health Alliance, Worcester, Massachusetts; ^4^Rhode Island Department of Health; ^5^New York State Department of Health; ^6^Division of Foodborne, Waterborne, and Environmental Diseases, National Center for Emerging and Zoonotic Infectious Diseases, CDC; ^7^Epidemic Intelligence Service, CDC.

Inadvertent ingestion of recreational waters contaminated with feces containing *Cryptosporidium* spp., an extremely chlorine-tolerant parasite, can result in gastrointestinal illness. In early 2023, a Massachusetts college notified the Massachusetts Department of Public Health (MDPH) that 19 of 50 (38%) members of the men’s and women’s swim teams had experienced diarrhea beginning 3 days after their return from a weeklong training trip to Puerto Rico. One ill swimmer reported receiving a positive ova and parasite test result for *Cryptosporidium*. On days 5 and 6 after return from Puerto Rico, symptomatic Massachusetts swimmers competed in two meets against New York and Rhode Island collegiate teams (meet 1 and meet 2, respectively), raising concern about the potential for secondary transmission.

Upon notifying MDPH of the ill swimmers (9 days after returning from Puerto Rico), ill Massachusetts swimmers were encouraged to submit stool specimens to the Massachusetts State Public Health Laboratory for testing with the BioFire FilmArray Gastrointestinal Panel.* A case in this investigation was defined as a gastrointestinal illness in a swim team member following the team’s travel to Puerto Rico. *Cryptosporidium*-positive specimens were forwarded to CDC and New York CryptoNet laboratories for molecular characterization (*1*,*2*). Swimmers with positive test results for *Cryptosporidium* were interviewed using a standardized questionnaire. On the same day as initial notification, MDPH notified the Puerto Rico, New York, and Rhode Island health departments; both the New York and Rhode Island health departments worked with their respective swim teams to identify secondary cases. At the same time, the Massachusetts college closed its swimming pool and hired a vendor to hyperchlorinate the pool water to inactivate *Cryptosporidium*.^† ^ This activity was reviewed by CDC and was conducted consistent with applicable federal law and CDC policy.§

Among the 19 symptomatic Massachusetts swimmers, 18 received testing, and stool specimens for 13 had positive test results for *Cryptosporidium* (Figure); the 13 patients ranged in age from 18 to 22 years, and eight were male. No hospitalizations occurred. Symptoms commenced 3 to 7 days after return to Massachusetts. Reported exposures to water sources while in Puerto Rico by the 13 patients with positive test results included the training pool (13), a waterfall (13), and the ocean (10). Swimmers with cryptosporidiosis were excluded from swimming activities until 2 weeks after resolution of diarrhea.¶

**FIGURE:**
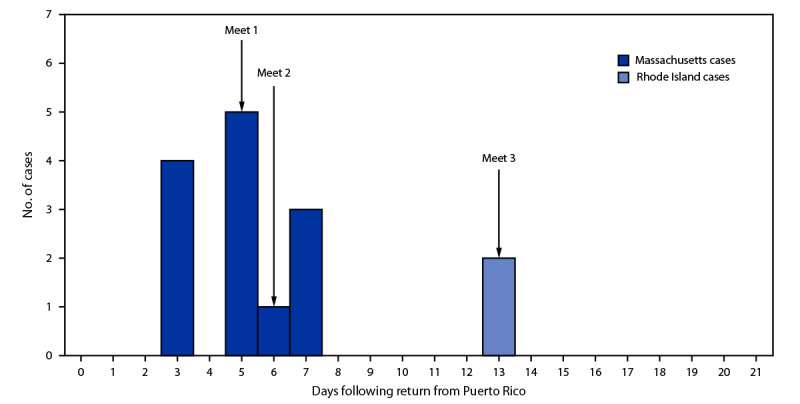
Cryptosporidiosis cases* among competitive collegiate swimmers at three swim meets, by state^†^ (N = 15) — Massachusetts and Rhode Island, 2023 * Confirmed by BioFire FilmArray Gastrointestinal Panel. https://www.biofiredx.com/products/the-filmarray-panels/filmarraygi/ ^†^ Thirteen cases in Massachusetts and two in Rhode Island.

No additional related cryptosporidiosis cases were identified or reported in Massachusetts or among potentially exposed New York swimmers at meet 1. Rhode Island officials reported that two swimmers became ill 7 days after meet 2 and received positive stool *Cryptosporidium* test results. Symptoms in these two swimmers began just after participating in another meet (meet 3) against another out-of-state university; no illnesses associated with that meet were reported. *Cryptosporidium parvum* IIaA16G3R1 was identified in specimens from five Massachusetts swimmers and IIaA17G2 and IIaA15G2R1 from one Massachusetts swimmer each. Subtype IIaA16G3R1 was also identified in specimens from the two Rhode Island swimmers, suggesting that secondary transmission occurred at meet 2.

This investigation highlights three important points. First, although there was no evidence of subsequent transmission from the Rhode Island swimmers, because of the regular intercollegiate competition and subsequent championship schedule, the potential exists for sustained *Cryptosporidium* transmission among competitive swimmers (*3*,*4*). Second, without the initial laboratory diagnosis of cryptosporidiosis in the Massachusetts swimmer, *Cryptosporidium* might not have been suspected and the pool might not have been immediately closed and disinfected, which could have led to further transmission, illustrating the importance of prompt testing of stool specimens from patients. Finally, there is an ongoing need to promote healthy swimming,** including recommendations for persons not to swim if they have diarrhea and to avoid swallowing swimming pool water to prevent waterborne disease.

## Footnotes

*
https://www.biofiredx.com/products/the-filmarray-panels/filmarraygi/

† CDC hyperchlorination recommendations in the Model Aquatic Health Code (MAHC) call for raising the free available chlorine (the active disinfectant form of chlorine) for a prolonged period to achieve 3-log_10_ (99.9%) *Cryptosporidium* inactivation (e.g., raising the free available chlorine to 20 ppm for 12.75 hours [MAHC 6.5.3.2]). www.cdc.gov/mahc

§ 45 C.F.R. part 46, 21 C.F.R. part 56; 42 U.S.C. Sect. 241(d); 5 U.S.C. Sect. 552a; 44 U.S.C. Sect. 3501 et seq.

¶ https://www.cdc.gov/parasites/crypto/gen_info/prevention-general-public.html (Accessed April 11, 2023).

** https://www.cdc.gov/healthywater/swimming/swimmers/rwi/diarrheal-illness.html (Accessed May 26, 2023).
